# Exploring Chemical Variability in the Essential Oil of *Artemisia absinthium* L. in Relation to Different Phenological Stages and Geographical Location

**DOI:** 10.1002/cbdv.202500743

**Published:** 2025-05-19

**Authors:** Paola Malaspina, Flavio Polito, Andrea Mainetti, Sana Khedhri, Vincenzo De Feo, Laura Cornara

**Affiliations:** ^1^ Department of Earth, Environment and Life Sciences (DISTAV) University of Genova Genova Italy; ^2^ Department of Pharmacy (DIFARMA) University of Salerno Fisciano Italy; ^3^ Biodiversity Service and Scientific Research, Gran Paradiso National Park Cogne Italy; ^4^ Laboratory of Management and Valorization of Forest Resources National Institute of Research on Rural Engineering Ariana Tunisia

**Keywords:** anatomy and micromorphology, Asteraceae, environmental conditions, phenological phase, phytochemistry

## Abstract

The plant essential oils (EOs) play a fundamental role in the adaptation to different habitats and environmental conditions. To verify the influence of these factors on the EO of *Artemisia absinthium* L., we conducted a pharmacognostic study on this species, collected during both the vegetative (V) and flowering (F) stages, in two different sites: Cogne (Northern Italy) and Ogliastro Cilento (Southern Italy). At the morphological level, only the shape of the leaf was affected, resulting in smaller and more deeply incised leaves in the Ogliastro samples. Considering the EOs, those from Cogne showed oxygenated monoterpenes as the main class of compounds in both non‐flowering (63.88%) and flowering (51.63%) stages, while those from Ogliastro had oxygenated sesquiterpenes (42.17%) as the main class of compounds in the non‐flowering stage, and monoterpene hydrocarbons (51.05%) in the flowering one. Moreover, the EOs of plants from the two regions showed qualitative differences; that is, elevated concentrations of myroxide and 3,6‐dihydrochamazulene were found in both Cogne samples, while camphor and davanone were found in Ogliastro‐V and β‐myrcene and β‐phellandrene were found in Ogliastro‐F. Overall, our data highlighted that both geographical locations and phenological stages influence the phytochemical profile of *A. absinthium* EOs, suggesting optimal harvesting conditions for maximizing certain bioactive compounds.

## Graphical abstract

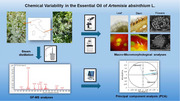

## 1 Introduction

Essential oils (EOs) are complex mixtures of secondary metabolites produced by numerous plant species, known for their high chemical variability and numerous applications in the pharmaceutical, food and cosmetic sectors [1]. In particular, many EOs are known to have significant inhibitory effects on microorganisms, and the new approach that uses the combinatory effect of EOs and their compounds with antibiotics has also revealed greater efficacy. Therefore, the use of EOs represents a new direction for the treatment of infections caused by bacteria, fungi and viruses [2]. Moreover, EOs are also well known for their antioxidant properties, which, by reducing oxidative stress on the skin, can be used in anti‐ageing products, as well as in aromatherapy applications [3].

In plants, EOs actively participate in all phenomena of adaptation to the environment and interaction with other organisms: defence against predators, attraction of pollinators, communication between species and many others [4]. Since these functions are linked to the relationship with the surrounding world, the variability in the chemical composition of EOs is the result of the pressures exerted on the plant by abiotic and biotic factors of a specific environment [5, 6]. This variability is evident even within plants of the same species that live in different environments and are subjected to different external stimuli. Variations in EOs chemical composition have therefore a crucial role in the survival and reproduction of plants in different ecosystems and conditions [6, 7]. Environmental conditions, geographical position, altitude and phenological stage of the plant are all factors that can influence the chemical composition of EOs [5, 6, 7]. Indeed, natural selection ensures the survival of plants whose EO composition has a higher adaptive value, as confirmed by studies carried out on *Tanacetum vulgare* L. growing in different environmental conditions [8].

In relation to the increasing demand of medicinal plants, different studies have been focused on cultivation strategy to improve chemical profile, biological properties and crop's productivity, in particular of species of Mediterranean habitat exposed to deficit irrigation [9] or of species growing in different latitudinal and altitudinal gradients, as in peripheral Alpine populations of *Lavandula angustifolia* Mill [10].

Understanding how these elements interact to modulate the chemical profile of EOs is useful to optimize cultivation and harvesting practices, as well as to enhance the biological and therapeutic properties of aromatic plants [11, 12]. During its life cycle, a plant goes through numerous phenological stages, characterized by different morphological, physiological and functional states, influenced by various biotic and abiotic factors. The phenological stage is crucial in the biosynthesis and accumulation of important metabolites, including those of the EOs, which can vary according to the requirements of the plants for life and growth [13]. For example, many monoterpenes and sesquiterpenes reach maximum concentrations during flowering, when the plant needs to attract pollinators or defend itself from predators and pathogens [14, 15]. Fluctuations in the levels of secondary metabolites in relation to phenological stages can affect chemical and functional characteristics of EOs [16, 17, 18, 19]. Geographical position and altitude, as well as the duration of exposure to the sun and UV radiation, variations in temperature and pressure and scarce availability of nutrients lead to the production of secondary metabolites useful to cope with extreme conditions [19, 20].

The goal of the present study was to valuate possible variations in the chemical composition of the EO of *Artemisia absinthium* L. in relation to different environmental conditions (Alpine and Mediterranean habitats), and different phenological stages (flowering and non‐flowering). *A. absinthium*, the absinthe, is a perennial herbaceous plant of the Asteraceae family, appreciated for its EO, which is a good candidate to be exploited in the pharmaceutical, food and cosmetic sectors for its antimicrobial, antioxidant and anti‐inflammatory properties [21]. In traditional Asian and European medicine, for a long time, it has been used for several therapeutic applications, such as in the treatment of gastrointestinal ailments and helminthiasis as well as fever and in addition, it has been a spice plant widely used as a valuable additive in the alcohol industry. Recent studies have also confirmed other biological properties of the compounds from this species, such as anti‐ulcer, hepatoprotective, analgesic, neuroprotective and many others [22].

It has been evaluated how phenological stages and geographical positions influence the chemical profile of EOs and, consequently, the potential applications of the plant. The results could be useful to study the optimal conditions for growing and harvesting the plant, with a view to a more correct management and valorisation of this important natural resource.

## 2 Results and Discussion

### 2.1 Macro‐Micromorphological Analyses

**FIGURE 1:**
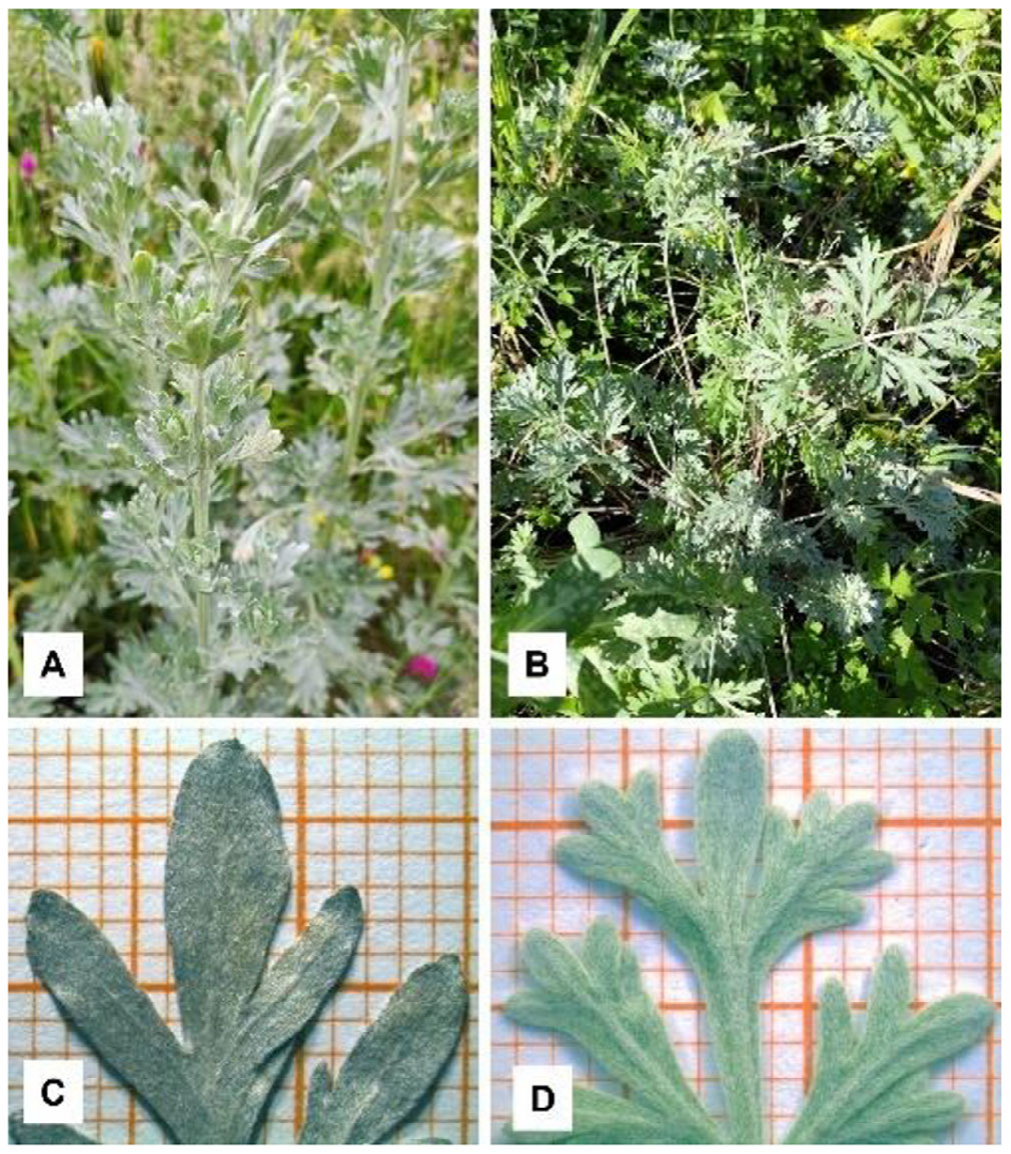
(A, C) Plant growing in Cogne (AO); (B, D) plant growing in Ogliastro Cilento (SA). (A, B) Plant in the field; (C, D) particular of the leaf.

*A. absinthium* collected from the two different sites (Figure 1A,B) showed different macromorphological traits exclusively in relation to the leaf shape. Indeed, the leaves of the plants from Cogne were larger, more developed, and more deeply incised than those from Ogliastro Cilento (Figure 1C,D).

**FIGURE 2:**
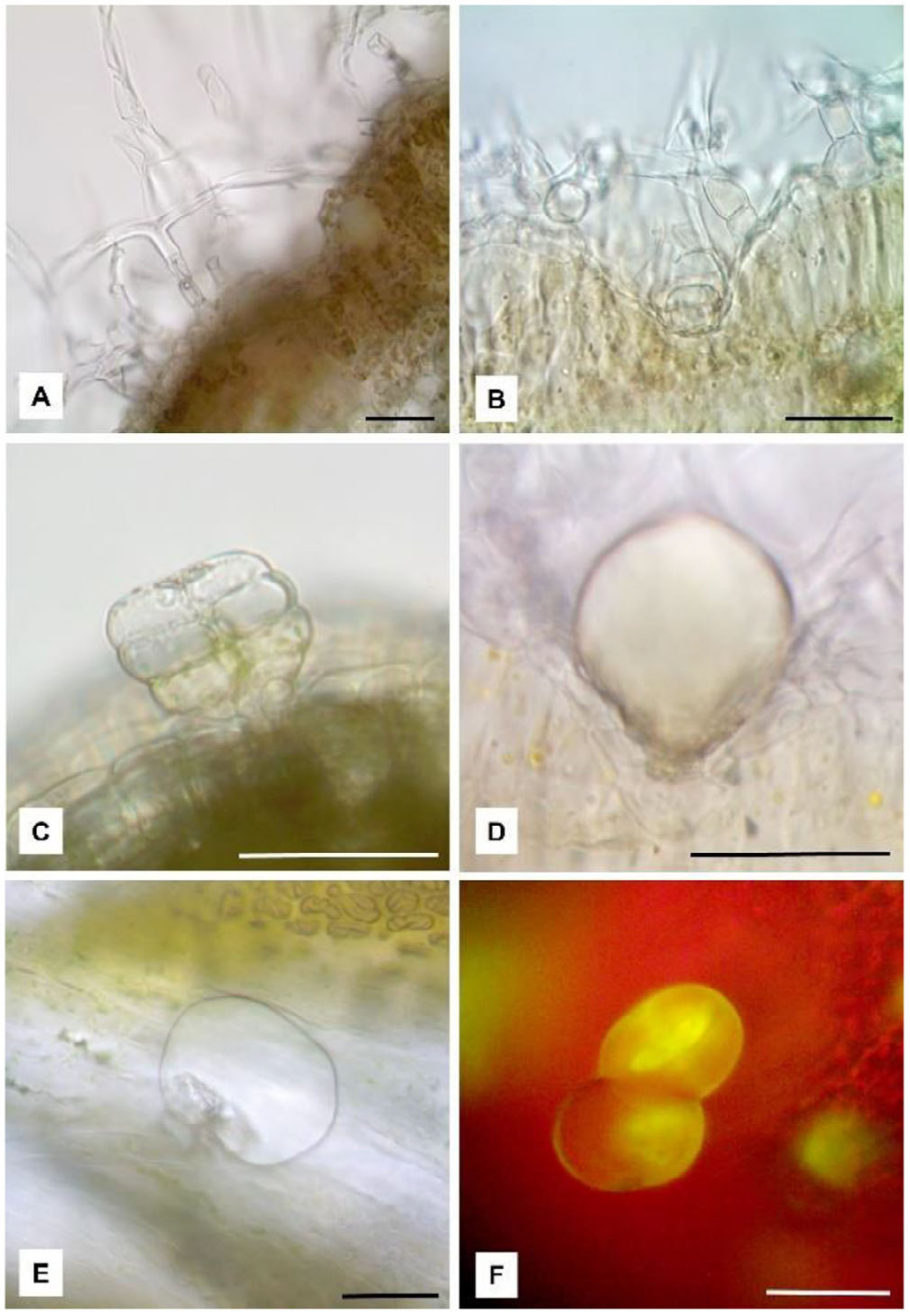
Light micrographs of *Artemisia absinthium* leaves from Ogliastro Cilento (A, D, E) and Cogne (B, C, F). (A) T‐shaped NGTs, formed by a multicellular stalk and an apical cell with two long arms; (B) a GT sunken in the epidermis and hidden by the NGTs; (C) a GT with the secretory cells organized in the biseriate structure; (D, E) GTs with the subcuticular space full of the EO; (F) GTs reacted positively with Fluorol Yellow 088. Bars = 50 micron.

On the contrary, no differences were found by anatomical and micromorphological observations. The leaves showed an isolateral, amphistomatous structure with a uniseriate epidermis and a palisade parenchyma formed by two layers of cells. As a distinctive feature, a dense tomentum of non‐glandular T‐shaped trichomes (NGTs) and glandular trichomes (GTs) was detected on both leaf surfaces. Each NGT was characterized by a multicellular stalk bearing an apical cell with long straight arms (Figure 2A). In leaf transversal section clarified with chloral hydrate, GTs appeared deeply embedded in the epidermis and hidden by the layer of NGTs (Figure 2B). Differently, on the corolla, the GTs protruded from the epidermal layer and they could be clearly observed since they were not hidden by the NGTs.

Some GTs showed their secretory cells arranged in the typical biseriate structure (Figure 2C), while in others the secretory cells were not more visible due to the raising of the cuticle and the filling of the subcuticular space with the EO (Figure 2D,E). The EO reacted positively with Fluorol Yellow 088, revealing the presence of lipophilic substances (Figure 2F).

**FIGURE 3:**
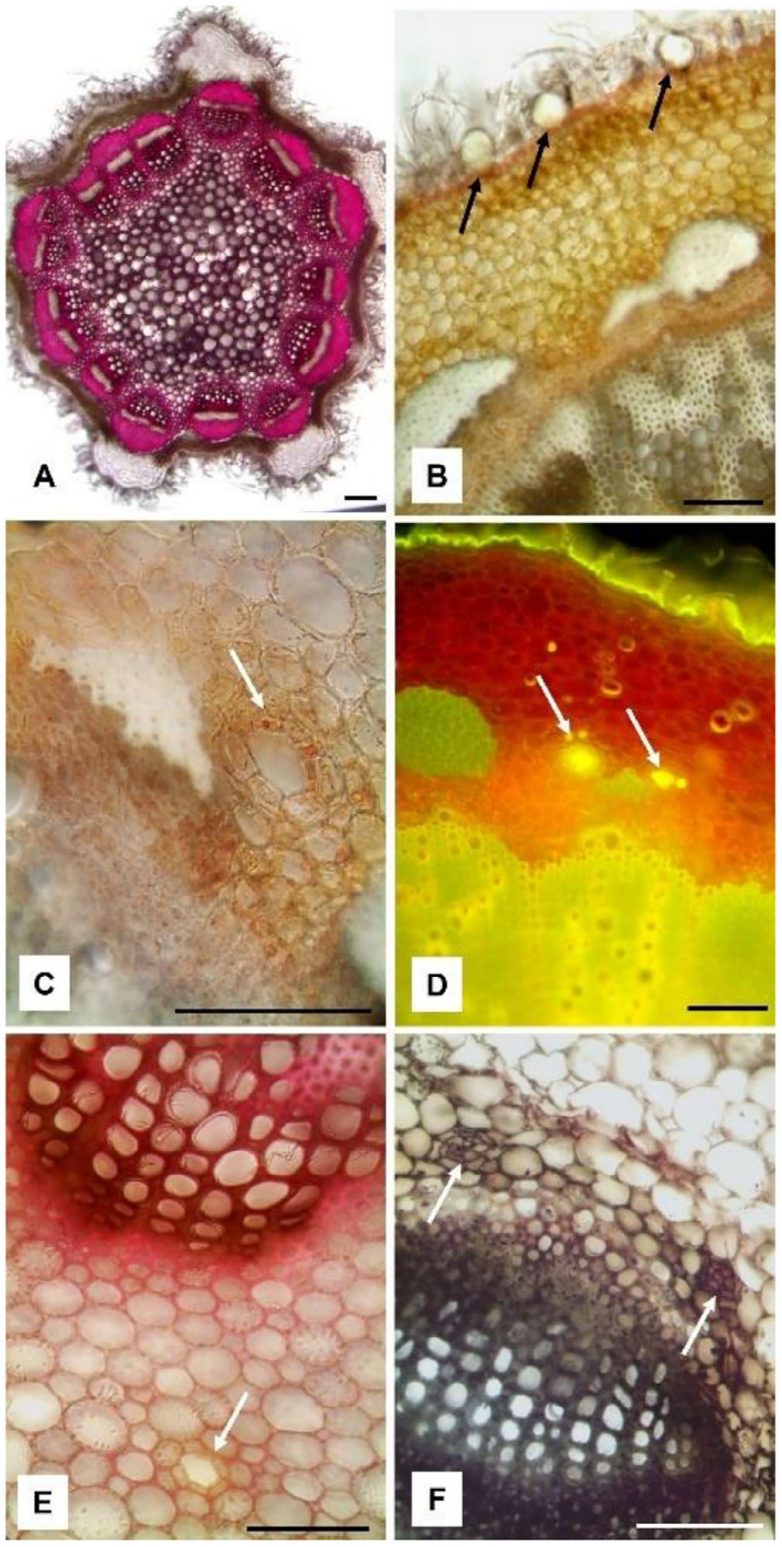
Light micrographs of *Artemisia absinthium* stem from Cogne (A, E) and Ogliastro Cilento (B–D) and of petiole from Ogliastro Cilento (F). (A) Transversal section stained with phloroglucinol‐HCl, with the prominent ribs well visible and the vascular bundles arranged in a circle; (B) transversal section cleared with chloral hydrate, showing GTs scattered among the NGTs (arrows); (C) transversal section showing a secretory duct (arrow) with small lipophilic drops stained by Sudan III; (D) transversal section stained by Fluorol Yellow 088, in which two secretory ducts are well visible due to the bright green‐yellow fluorescence of the secretion (arrows); (E) transversal section stained with phloroglucinol‐HCl, where a duct (arrow) is present in the pith close to the xylematic portion; (F) transversal section of the petiole stained by Sudan Black, where two secretory ducts are visible near the xylematic portion of the bundle (arrows). Bars = 100 micron.

In transversal section, the stem showed an irregular pentagonal shape, with prominent ribs formed by collenchymatous tissue separated by chlorenchyma (Figure 3A). The entire surface of the stem was covered by a dense layer of NGTs, among which numerous GTs could be observed (Figure 3B, arrows). GTs appeared raised from the epidermis and they were formed by a short neck bearing a big glandular head, similarly to those present on the leaves. In the stem, the vascular bundles were arranged in a circle and separated from each other by the parenchymatous tissue (Figure 3A,B). In the cortical layer of the stem, at the side of the caps of sclerenchyma covering the vascular bundles, some secretory ducts were found (Figure 3C,D). Small lipophilic drops inside the duct secretory cells reacted positively with Sudan III (Figure 3C, arrow) and the secretion within the duct was green‐yellow stained by Fluorol Yellow 088, highlighting the presence of terpenoids (Figure 3D, arrows). Other secretory ducts were also observed in the pith close to the xylematic portion (Figure 3E, arrow). In the petiole transversal section, after staining with Sudan Black, two small secretory ducts were identified near the xylematic portion of the midrib (Figure 3F, arrows).

**FIGURE 4:**
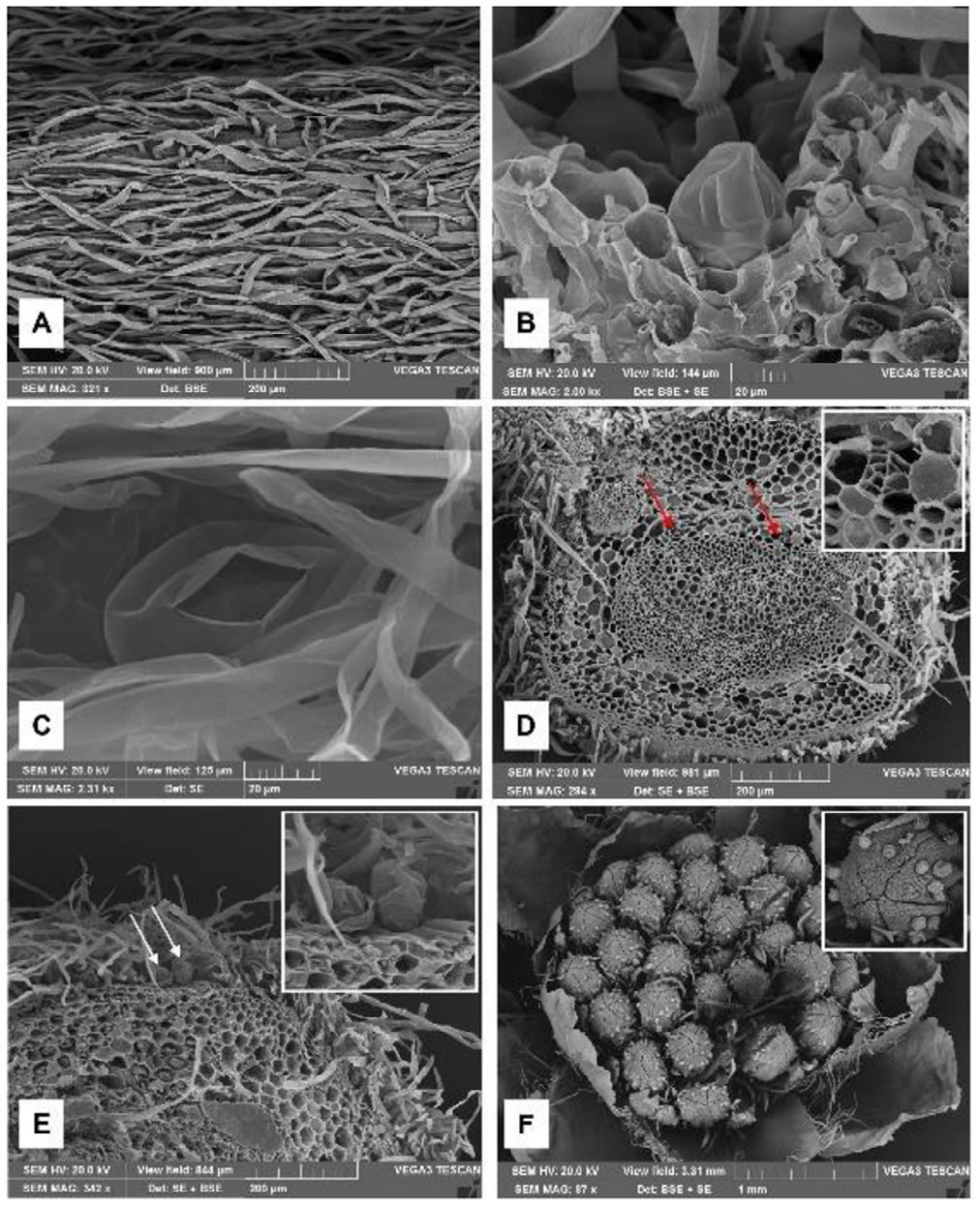
SEM micrographs of *Artemisia absinthium*. (A) Leaf surface covered by a dense layer of T‐shaped NGTs; (B) leaf transversal section, showing a GT sunken in the epidermis with a short neck and a bladder head; (C) view of the leaf surface, where a GT with a polygonal apical opening in the cuticle is visible; (D) petiole transversal section, showing two secretory ducts (arrows and magnification in the frame); (E) stem transversal section, showing GTs hidden by the dense tomentum of NGTs (arrows and magnification in the frame); (F) inflorescence, where several GTs are present on the corolla of each flower (magnification in the frame).

Scanning electron microscopy (SEM) observations confirmed previous data and allowed us to better characterize the micromorphological and anatomical features of *A. absinthium*. The leaf surface appeared covered by a dense layer of T‐shaped NGTs (Figure 4A), each one showing two flattened, long and thin arms. These trichomes made it difficult to observe the characteristics of the underlying epidermis. On the contrary, in cross‐section, it was possible to observe the presence of several GTs hidden by NGTs and embedded in the epidermis. GTs were formed by a short neck and a circular‐bladder glandular head, covered by a thin cuticle (Figure 4B). In addition, by increasing the magnification on the leaf surface, it was possible to observe several GTs in which the cuticle had broken through the formation of a polygonal apical opening that allowed distinguishing the underlying secretory cells (Figure 4C). Through this opening, the secretion is released outside.

SEM analysis also revealed the presence of two secretory ducts in the petiole near the xylematic portion of the midrib of the leaf (Figure 4D, arrows and frame). Furthermore, several GTs were identified in the stem, hidden by the dense tomentum of NGTs and protruding from the epidermis (Figure 4E, arrows and frame). Finally, considering the inflorescence, several GTs were observed on the corolla of each flower (Figure 4F and frame).

### 2.2 Yield of the EOs

Table 1 shows the percentage yields obtained from the distillation processes. The letter ‘V’ indicates plants collected during the vegetative stage, while the letter ‘F’ indicates plants collected during the flowering stage (blooming). The highest yield was obtained with the EO of *A. absinthium* from Cogne‐V (0.33%). It should be noted that the EO yield of Cogne‐F was lower than that obtained from Cogne‐V. On the contrary, the plants from Ogliastro Cilento showed smaller differences between the two phenological stages: 0.24% for Ogliastro‐V and 0.27% for Ogliastro‐F.

**TABLE 1:** EOs quantity and yield.

Sample	Distilled quantity (g)	EO obtained (g)	Yield %
*Artemisia absinthium* Cogne‐V	860.51	2.84	0.33
*A. absinthium* Cogne‐F	410.63	0.87	0.21
*A. absinthium* Ogliastro‐V	576.60	1.36	0.24
*A. absinthium* Ogliastro‐F	2426.23	6.55	0.27

### 2.3 Chemical Composition of the EOs

The composition of the EOs is reported in Table 2. Niney‐three compounds were identified: 29 in the EO of Cogne‐V (99.75% of the total), 52 in the EO of Cogne‐F (99.67%), 32 in the EO of Ogliastro‐V (99.63%) and 42 in the of EO Ogliastro‐F (99.98%).

**TABLE 2:** Composition of the EOs.

N.	Compound	%				Kia	Kib	Identificationc
		Cogne‐V	Cogne‐F	Ogliastro‐V	Ogliastro‐F			
**1**	6‐Methyl‐1‐heptene	—	—	—	0.31	804		1, 2, 3
**2**	α‐Pinene	—	0.51	1.43	0.49	862	1025	1, 2, 3
**3**	Camphene	—	0.23	1.81	1.11	874	1068	1, 2, 3
**4**	Isobutyl butanoate	—	—	0.51	—	878	1174	1, 2
**5**	4(10)‐Thujene	1.27	1.08	—	—	900	1115	1, 2
**6**	β‐Phellandrene	—	—	—	21.22	902	1209	1, 2
**7**	1‐Octen‐3‐ol	—	0.2	—	0.50	908	1452	1, 2
**8**	β‐Myrcene	0.60	1.19	3.07	17.78	920	1161	1, 2, 3
**9**	α‐Phellandrene	0.65	—	—	7.05	928	1168	1, 2, 3
**10**	α‐Terpinene	—	—	0.72	0.27	938	1178	1, 2, 3
**11**	*p*‐Cymene	—	0.32	—	1.55	946	1270	1, 2, 3
**12**	Limonene	—	—	0.59	—	950	1198	1, 2, 3
**13**	3‐Carene	—	—	—	0.34	951	1147	1, 2
**14**	Eucalyptol	0.46	0.30	2.41	0.23	953	1211	1, 2, 3
**15**	*trans*‐β‐Ocimene	0.67	1.72	—	0.79	965	1250	1, 2
**16**	Fenchone	—	0.38	—	—	966	1400	1, 2, 3
**17**	γ‐Terpinene	—	0.18	1.26	0.45	979	1245	1, 2, 3
**18**	α‐Pinene oxide	0.96	0.52	—	—	1020	1364	1, 2
**19**	Linalool	—	—	3.04	5.07	1020	1543	1, 2, 3
**20**	*cis*‐Thujone	—	0.23	—	—	1023	1423	1, 2, 3
**21**	Nonanal	—	—	—	0.23	1025	1392	
**22**	*trans*‐Thujone	2.5	1.89	—	0.98	1032	1440	1, 2, 3
**23**	*cis*‐(−)‐1,2‐Limonene oxide	0.48		—	—	1035	1458	1, 2
**24**	*allo*‐Ocimene		0.19	—	—	1046	1367	1, 2
**25**	Myroxide	44.41	41.28	—	7.24	1057	1484	1, 2
**26**	Camphor	—	—	19.07	—	1059	1515	1, 2, 3
**27**	Borneol	—	—	0.59	—	1076	1700	1, 2, 3
**28**	*cis*‐Chrysanthenol	0.91	1.96	—	0.65	1080	1762	1, 2
**29**	Lavandulol	—	0.18	—	0.5	1082	1679	1, 2
**30**	*p*‐Mentha‐1,5‐dien‐8‐ol	—	0.68	—	—	1085	1670	1, 2
**31**	3‐*p*‐Menthen‐7‐al	1.58	—	—	—	1087	1568	1, 2
**32**	Terpinen‐4‐ol	—	0.10	2.16	0.57	1088	1601	1, 2
**33**	2‐Methyl isoborneol	—	0.10	—	—	1091		1, 2
**34**	2,6‐Dimethyl‐3,7‐octadiene‐2,6‐diol	—	1.22	—	—	1095	1936	1, 2
**35**	α‐Terpineol	—	—	0.50	0.28	1099	1694	1, 2, 3
**36**	Nerol	—	0.10	—	—	1135	1795	1, 2
**37**	*n*‐Hexyl 2‐methylbutanoate	—	0.11	—	—	1146	1438	1, 2
**38**	*cis*‐Chrysanthenyl acetate	0.56	—	—	—	1169	1561	1, 2
**39**	Perilla aldehyde	—	0.11	—	—	1175	1794	1, 2
**40**	*p*‐Mentha‐1,8‐dien‐7‐al	—	—	—	0.25	1179		1, 2
**41**	Bornyl acetate	—	—	2.64	—	1189	1579	1, 2
**42**	*trans*‐Sabinyl acetate	3.69	2.91	—	—	1192	1643	1, 2
**43**	Lavandulol acetate	—	—	—	0.46	1193	1602	1, 2
**44**	Thymol	1.36	—	2.44	—	1195	2164	1, 2, 3
**45**	Carvacrol	1.26	0.44	1.23	—	1202	2211	1, 2, 3
**46**	Myrcenylacetate	—	—	0.52	—	1216		1, 2
**47**	Ethyl phenyl propanoate	—	—	0.35	—	1246	1914	1, 2
**48**	Eugenol	—	0.12	—	—	1254	2163	1, 2, 3
**49**	Neryl acetate	—		—	0.66	1268	1718	1, 2
**50**	α‐Copaene	—	0.52	—	—	1269	1491	1, 2
**51**	β‐Bourbonene	—	0.33	—	—	1277	1523	1, 2
**52**	β‐Cubebene	—	0.13	—	—	1283	1460	1, 2
**53**	β‐Elemene	—	0.27	—	0.26	1286	1591	1, 2
**54**	*iso*‐Italicene	—	0.11	—	—	1294	1490	1, 2
**55**	*trans*‐Caryophyllene	3.65	4.20	0.83	2.81	1307	1598	1, 2
**56**	β‐Copaene	—	0.11	—	—	1313	1580	1, 2
**57**	Lavandulyl butyrate	—		—	0.71	1319		1, 2
**58**	Sesquisabinene	—	0.21	—	0.26	1331	2081	1, 2
**59**	α‐Humulene	—	0.44	—	0.32	1337	1667	1, 2
**60**	Neryl propionate	—	—	—	0.23	1349	1778	1, 2
**61**	Ethyl cinnamate	—	—	2.55	—	1353	2081	1, 2
**62**	1,2‐Dehydrosesquicineole	—	0.14	—	—	1357	1823	1, 2
**63**	Germacrene D	7.08	4.79	0.61	0.48	1365	1708	1, 2
**64**	γ‐Curcumene	—	2.19	—	2.81	1367	1692	1, 2
**65**	β‐Selinene	—	—	0.46	—	1369	1717	1, 2
**66**	γ‐Himachalene	—	—	—	0.64	1373	1709	1, 2
**67**	Phenyl ethyl 2‐methylbutanoate	0.44	0.10	—	—	1374	1988	1, 2
**68**	Bicyclogermacrene	—	0.24	—	—	1380	1734	1, 2
**69**	Neryl isobutanoate	—	—	—	1.49	1383	1780	1, 2
**70**	Geranyl isobutanoate	0.93	0.30	—	0.75	1385	1819	1, 2
**71**	Davana ether	—	—	2.20	—	1397		1, 2
**72**	3,6‐Dihydrochamazulene	10.75	17.48	—	7.36	1398		1, 2
**73**	*trans*‐Nerolidol	—	—	4.26	—	1449	2036	1, 2
**74**	Germacrene D‐4‐ol	0.47	—	—	—	1457	2057	1, 2
**75**	Caryophyllene oxide	—	—	0.83	0.24	1458	1986	1, 2
**76**	Geranyl 2‐methylbutanoate	1.70	1.10	—	3.46	1464	1902	1, 2
**77**	(E)‐Farnesene epoxide	0.57	0.80	—	—	1475		1, 2
**78**	Davanone	—	—	35.88	—	1476	2040	1, 2
**79**	(R)‐lavandulyl (R)‐2‐methylbutanoate	1.26	0.17	—	—	1482		1, 2
**80**	Geranyl isovalerate	—	—	0.40	—	1491	1893	1, 2
**81**	Caryophylla‐4(12),8(13)‐dien‐5β‐ol	—	0.98	—	—	1498	2316	1, 2
**82**	β‐Eudesmol	—	—	1.20	—	1517	2238	1, 2
**83**	Methyl jasmonate	—	—	1.00	—	1520		1, 2
**84**	*epi*‐γ‐Eudesmol	1.52	0.66	—	0.73	1530	2106	1, 2
**85**	α‐Bisabolol	1.48	0.87	—	—	1560	2214	1, 2
**86**	Zingiberenol	—	—	—	0.55	1565	2109	1, 2
**87**	Chamazulene	0.76	0.34	3.58	—	1598	2430	1, 2
**88**	Methyl isocostate	—	—	0.44	—	1662		1, 2
**89**	(Z)‐β‐Curcumen‐12‐ol	6.40	1.9	—	—	1805		1, 2
**90**	Pentyl‐curcumene	—	3.04	—	7.64	1848	1786	1, 2
**91**	Geranyl‐*p*‐cymene	—	—	1.05	—	1851		1, 2
**92**	Phytol	—	—	—	0.26	1955	2622	1, 2
**93**	*trans*‐Geranyl.geraniol	1.38	—	—	—	2047		1, 2
	**Total**	99.75	99.67	99.63	99.98			
	**Monoterpene hydrocarbons**	3.19	5.23	8.88	51.05			
	**Oxygenated monoterpenes**	63.88	51.63	33.60	16.89			
	**Sesquiterpene hydrocarbons**	11.49	13.88	5.48	8.29			
	**Oxygenated sesquiterpenoids**	21.19	22.69	42.17	8.88			
	**Others**	5.71	6.24	9.50	14.87			

Abbreviation: —, absent.

^a^ The Kovats retention indices are relative to a series of *n*‐alkanes (C10–C35) on the apolar DB‐5.

^b^ The Kovats retention indices are relative to a series of *n*‐alkanes (C10–C35) on the polar HP Innowax capillary columns.

^c^ Identification method: 1 = comparison of the Kovats retention indices with published data, 2 = comparison of mass spectra with those listed in the NIST 02 and Wiley 275 libraries and with published data, and 3 = co‐injection with authentic compounds.

The distribution of the chemical classes in the EOs from Cogne (Northern Italy) was very similar between non‐flowering and flowering stages: the main class in both was that of oxygenated monoterpenes (63.88% for EO Cogne‐V and 51.63% for EO Cogne‐F), followed by oxygenated sesquiterpenes (21.19% for EO Cogne‐V and 22.69% for EO Cogne‐F). Even the minority classes of sesquiterpene hydrocarbons and monoterpene hydrocarbons had similar percentages between the two stages (11.49% vs. 13.88% and 3.19% vs. 5.23%, respectively). The percentages of compounds not belonging to these classes were also similar (5.71% for EO Cogne‐V and 6.24% for EO Cogne‐F). Considering the main components, also in this case, the EOs Cogne‐V and Cogne‐F showed similarity: myroxide (*cis*‐epoxy‐ocimene) was the main component (44.41% vs. 41.28%), followed by 3,6‐dihydrochamazulene (10.75% vs. 17.48%).

The comparison between the EOs from Ogliastro Cilento (Southern Italy) showed a different composition between the non‐flowering stage and the flowering one. In Ogliastro‐V, the main class was that of oxygenated sesquiterpenes (42.17%), followed by oxygenated monoterpenes (33.60%), monoterpene hydrocarbons (8.88%) and sesquiterpene hydrocarbons (5.48%). In Ogliastro‐F, the main class was that of monoterpene hydrocarbons (51.05%), followed by oxygenated monoterpenes (16.89%), oxygenated sesquiterpenes (8.88%) and sesquiterpene hydrocarbons (8.29%). The percentages of compounds not belonging to these classes were also different (9.50% for EO Ogliastro‐V and 14.87% for Ogliastro‐F). In addition, the main components were different. For the EO Ogliastro‐V, the main component was davanone (35.88%), followed by camphor (19.07%), while in the EO Ogliastro‐F, the main component was β‐phellandrene (21.22%), followed by β‐myrcene (17.78%).

### 2.4 Principal Component Analysis and Hierarchical Cluster Analysis on the Chemical Compositions

**FIGURE 5:**
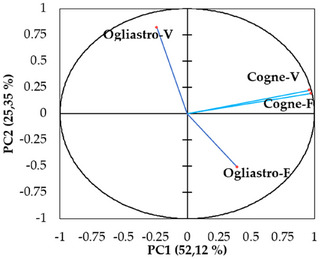
Principal component analysis (PCA) conducted on the chemical composition of the four EOs (Cogne‐V, Cogne‐F, Ogliastro‐V, Ogliastro‐F).

**FIGURE 6:**
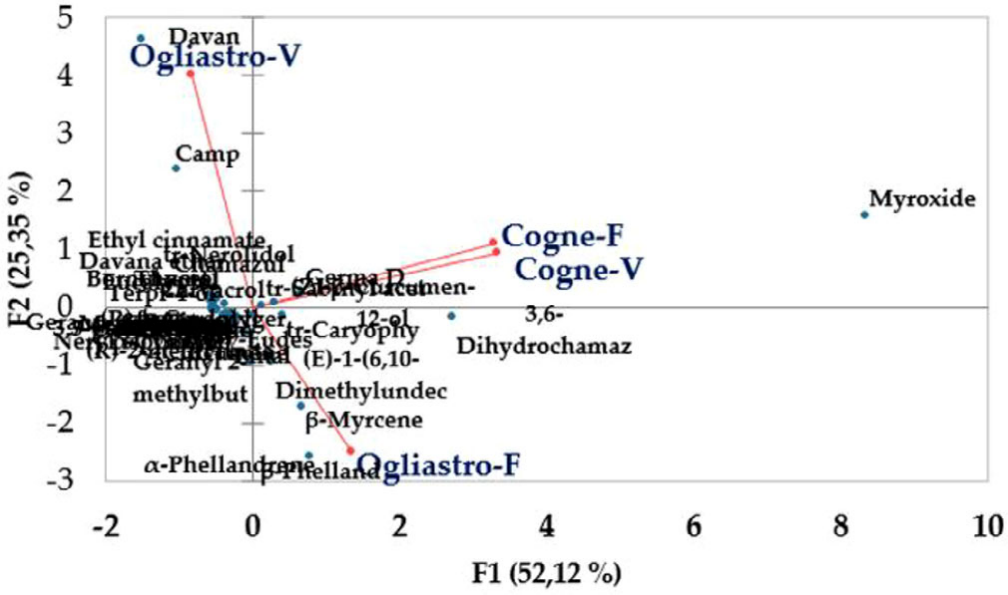
Principal component analysis (PCA) conducted on the chemical compositions of the four EOs (Cogne‐V, Cogne‐F, Ogliastro‐V, Ogliastro‐F); mapping variables and observations in principal component space.

**FIGURE 7:**
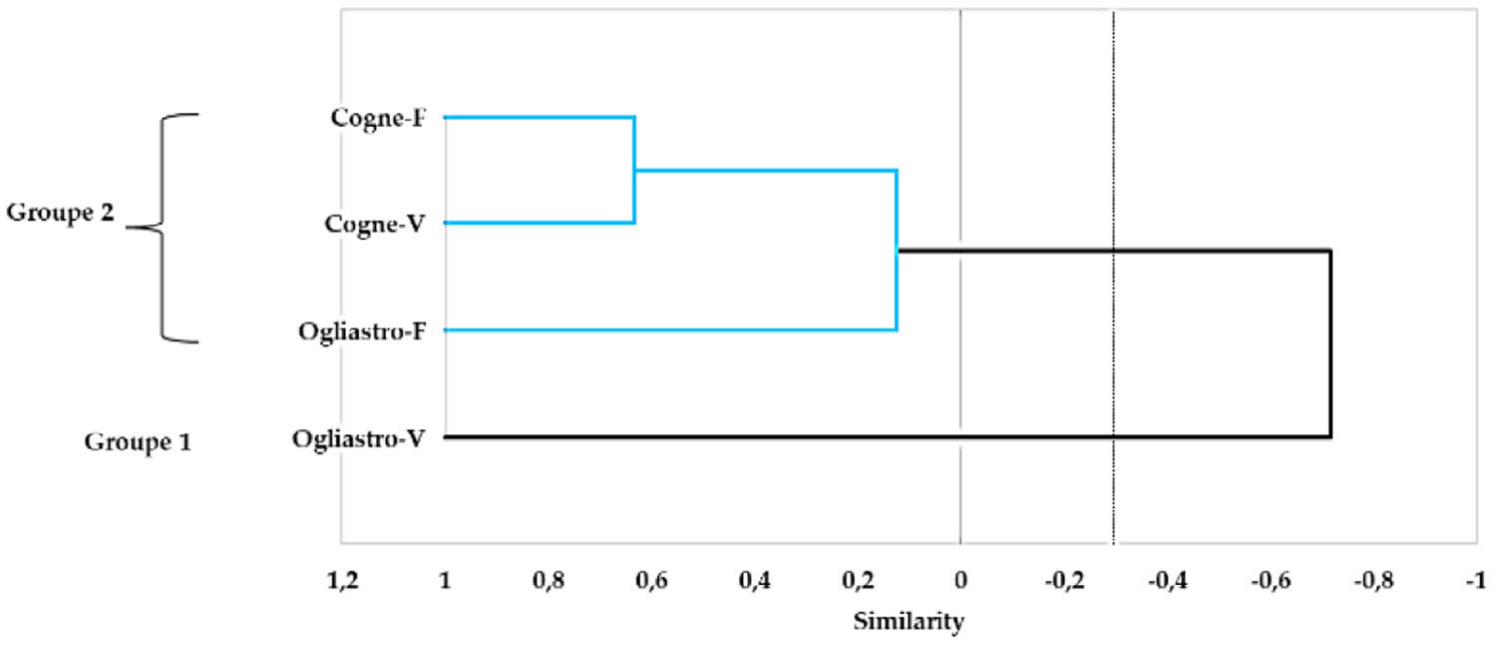
Hierarchical cluster analysis (HCA) conducted on the chemical composition of the four EOs (Cogne‐V, Cogne‐F, Ogliastro‐V, Ogliastro‐F).

The principal component analysis (PCA) conducted on the chemical composition of the four EOs revealed a distinct separation between the samples based on geographical locations and development stages. The first two principal components (PC1 and PC2) explained 52.12% and 25.35% of the total variance, respectively, accounting for a combined 77.47% of the total variability in the dataset (Figure 5). EOs from Cogne were predominantly clustered on the positive side of the PC1 axis, while those from Ogliastro Cilento were distributed on the negative side. This fact indicates a strong geographic influence on the chemical composition of the EOs. In addition, EOs sourced from different developmental stages within the Cogne location demonstrated a positive correlation, marked by elevated concentrations of specific components, including myroxide and 3,6‐dihydrochamazulene. In contrast, the chemical profiles of the EOs from Ogliastro Cilento exhibited distinct differences, with notable increases in compounds such as camphor and davanone in Ogliastro‐V and in compounds such as β‐myrcene and β‐phellandrene in Ogliastro‐F (Figure 6). This observation was further confirmed by the hierarchical cluster analysis (HCA) (Figure 7), which reveals that the EO Ogliastro‐F was separate from the other three EOs.

### 2.5 Similarity Percentage Analysis

**FIGURE 8:**
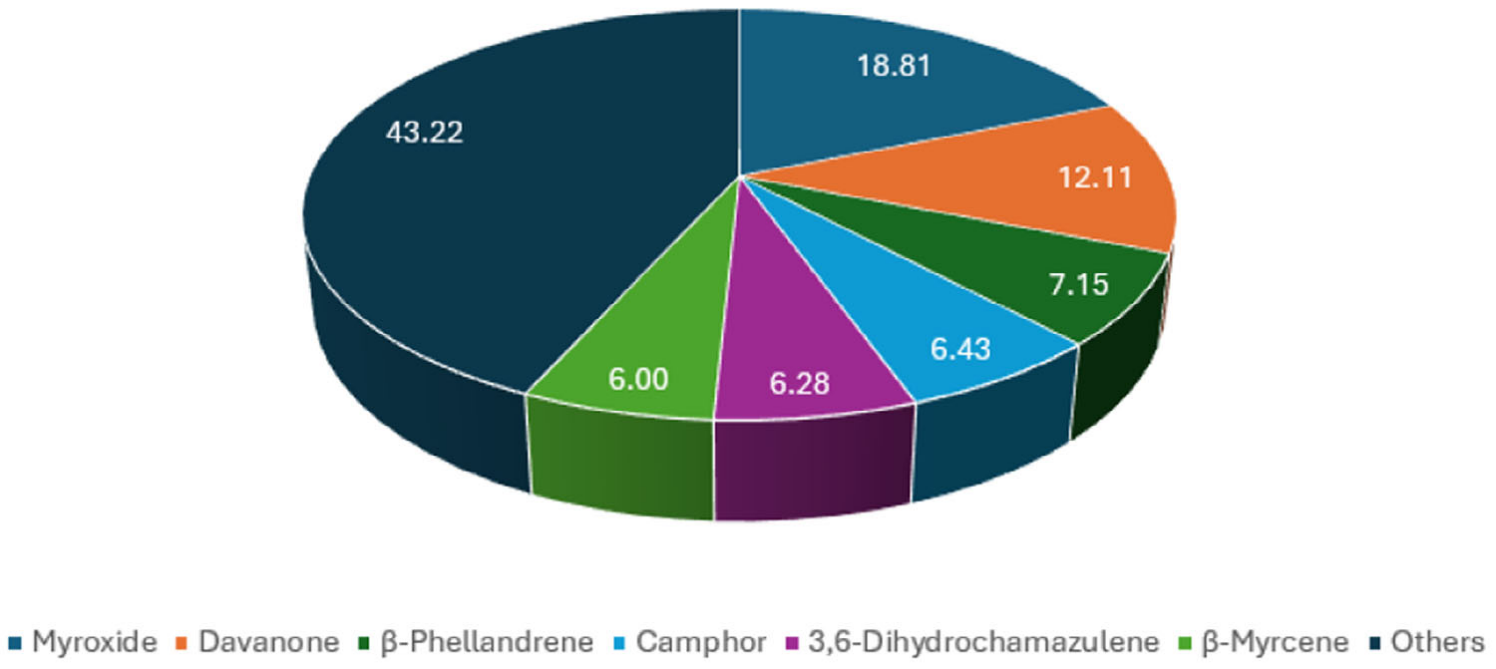
Percentage of contribution of the most important compounds to the total difference between the four EOs (Cogne‐V, Cogne‐F, Ogliastro‐V, Ogliastro‐F).

The pie chart in Figure 8 shows the results of the statistical analysis performed using the Similarity Percentage (SIMPER) test. It summarizes the compounds that are mainly responsible for the differences between the four EOs, reporting for each of them the percentage of contribution to the total difference. Myroxide, davanone and β‐phellandrene are the compounds that give the greatest contribution to diversity, with percentages of contribution of 18.81%, 12.11% and 7.15%, respectively. These are followed by camphor (6.43%), 3,6‐dihydrochamazulene (6.28%) and β‐myrcene (6.00%). All together, these components are responsible for 56.78% of the differences. The remaining 43.22% is given by the sum of the percentages of the other components (all less than 6.00%). With only one replicate per group, the SIMPER analysis performed has only exploratory and descriptive value. The complete results of the SIMPER test are included in the Supporting Information.

The variations in leaf phenotype observed between *A. absinthium* from Cogne and Ogliastro Cilento may represent an adaptation of plants to different habitats, thus reflecting survival strategies evolved in response to specific environmental factors, such as temperature, altitude and solar radiation. In particular, leaf size may play an important role in leaf thermal regulation. In Mediterranean climate characterized by hot, sunny and dry summers (as in Southern Italy), small leaves can be a favourable trait to preserve a low leaf temperature and to avoid overheating. In addition, leaf size tends to decrease with decreasing water availability to reduce water loss [23, 24]. On the contrary, leaves tend to be bigger in habitats with summer characterized by warmer temperatures and abundant precipitation, such as in the Alpine climate. Therefore, the differences observed between leaves of *A. absinthium* sampled in different areas may be due to adaptive strategies to different environmental conditions.

In general, the plants of *A. absinthium* from the two study areas shared the same micromorphological characteristics typical of other species of the same genus, particularly the presence of both non‐glandular and glandular trichomes (NGTs and GTs, respectively) covering all the aerial parts of the plant. These epidermal structures play a fundamental role in enabling the plant to interact with its environment and respond to stress factors. The presence of a thick coat of NGTs has a key role in limiting perspiration, reflecting solar radiation, controlling water loss and regulating temperature. In addition, the secretion of EO released by GTs into the environment is important for the plant's reproductive strategies and defence against herbivores and pathogens [25, 26, 27].

In agreement with the study of Janaćković et al. [28], *A. absinthium* showed the leaf epidermis covered by a dense tomentum made up of T‐shaped NGTs and biseriate GTs. As previously reported for the leaf of other *Artemisia* [29], GTs are hidden under the dense and uniform layer of NGTs, and they are embedded in epidermal depressions. As previously reported [28], the leaf blade of *A. absinthium* was amphistomatous, with a two‐layer palisade, a prominent midvein with two lateral ribs, and it showed the absence of secretory ducts in the mesophyll. On the contrary, in the petiole as well as in the stem, small secretory ducts were detected in the cortex and/or in the pith.

Considering the yield and composition of EO from *A. absinthium*, different reviews [6, 21, 30] reported that this parameter varies from 0.12% to 0.51%, in line with what was found by us. In the above studies, the chemical composition showed as the main components most frequently found α‐ and β‐thujone, camphor, camphene, α‐pinene, *p*‐cymene, 1,8‐cineole, β‐myrcene, linalool, bornyl acetate, β‐phellandrene, α‐terpineol, α‐terpinene, 1,4‐terpeniol, myroxide, sabinyl acetate, cadinene, guaiazulene, caryophyllene oxide, caryophyllene, *cis*‐chrysanthenol, *trans*‐sabinyl acetate and chamazulene. They are also referred to as the main chemotypes: sabinene, myrcene, α‐ and β‐thujone, myroxide and sabinyl acetate types and also ‘mixed’ ones [6, 21, 22, 30, 31]. However, more than 20 chemotypes of *A. absinthium* have been described in the literature [6].

Most of the components reported were also found by us: β‐phellandrene and camphor resulted as major components, while others (e.g., α‐ and β‐thujone, camphor, α‐pinene, camphene, β‐myrcene, eucalyptol, linalool, α‐terpineol, chamazulene) were present occasionally and never as the major components. We found different chemotypes: EOs from Cogne belong to the myroxide chemotype, while the EOs from Ogliastro Cilento were a mixed chemotype.

To define the plant chemotype, an important role is played by the development stages, which affect the accumulation and change of secondary compounds [32, 33, 34, 35, 36]. Nguyen et al. [37] showed how different factors could influence the content and composition of EO in *A. absinthium*, for example, different country of origin, ontogenetic factors (development stages), morphogenetic factors (plant portion used), environmental factors and extraction methods are all involved in the variability of EO constituents. For example, during plant development it was observed mainly a quantitative variation in the phytochemical profile of the EO in *A. absinthium* from Lithuania and Spain, even though concerning different compounds [38, 39]. These studies demonstrated how the chemical profile of the EO, particularly the distribution and the amounts of the main components, can vary in relation to the harvesting periods and phenological stages. This fact can be considered to choose the appropriate harvest time to obtain the most favourable constituents for food, pharmaceutical and herbal products [39, 40, 41].

In our study both the origin of plant and its phenological stage can determine phytochemical variations: mainly quantitative fluctuations were detected in the EOs from Cogne, while both quantitative and qualitative in the EOs from Ogliastro Cilento. Considering the two different phenological stages within the same place of origin, it can be observed that *A. absinthium* from Cogne almost completely conserved the compositional characteristics of the EOs, while *A. absinthium* from Ogliastro Cilento showed notable differences.

In addition, the comparison between the two different phenological stages of the two locations (EO Cogne‐V vs. Ogliastro‐V and EO Cogne‐F vs. Ogliastro‐F) showed important differences between the EOs both in the type of components and in their quantities. It even happens that the main components of the EO Cogne‐V are not present in the EO Ogliastro‐V and vice versa. During the flowering stage, this happens only for β‐phellandrene (present in EO Ogliastro‐F but not in EO Cogne‐F), while the other main components are present in both EOs, albeit in very different quantities.

Also, environmental conditions, including altitude, significantly influence the phytochemical profile of plants, especially aromatic ones [42, 43, 44, 45]. Many studies highlight how altitude plays a determining role in the variations of the composition of EOs, also within the same species [46, 47, 48, 49]. Such variations may also influence the biological activities of the EOs, as reported in *Satureja thymbra* L. which, growing at low altitudes, presented a thymol chemotype with high antifungal activity, while growing at high altitudes showed a carvacrol chemotype with high antibacterial activity [47]. As further proof, also *A. absinthium* collected by us in sites characterized by different environmental conditions, including altitude, showed variations in both qualitative and quantitative characteristics of the EO, which could influence its biological properties [50, 51, 52]. In fact, temperature can significantly influence the characteristics of EOs, both qualitatively and quantitatively. In general, higher temperatures induce a greater production of EOs, thanks to a greater evaporation of volatile compounds that induces the plant to remedy this event by increasing their production. High temperatures also stimulate the production of lighter and more volatile compounds, which can evaporate more easily and carry out their numerous activities: among these, monoterpenes, esters and aldehydes. Milder temperatures instead cause a greater production of more stable and less volatile molecules such as sesquiterpenes, terpenoids and alcohols [53, 54, 55]. This trend was found in the two EOs from Ogliastro Cilento. The EO Ogliastro‐V was obtained from a plant harvested in May, a month in which the temperatures are warm but not excessively (average temperatures of 18°C–20°C). This EO was rich in compounds belonging to the class of oxygenated sesquiterpenes (42.17%) such as davanone (35.88%), heavier and oxygenated and phenolic compounds such as linalool (3.04%), camphor (19.07%), terpinen‐4‐ol (2.16%), bornyl acetate (2.64%), thymol (2.44%) and carvacrol (1.23%). Conversely, the EO Ogliastro‐F was collected in the July, a typically much warmer month and its composition was particularly rich in light compounds: monoterpene hydrocarbons (51.05%) and oxygenated monoterpenes (16.89%) such as β‐phellandrene (21.22%) and β‐myrcene (17.78%). The variations are less accentuated in the Lillaz samples which were collected in months (May and June) with very similar conditions and characterized by cooler temperatures (average temperatures between 6°C and 18°C). Cooler temperatures were reflected in a lower production of monoterpenes (especially hydrocarbons) and in a higher production of sesquiterpene hydrocarbons. The average annual rainfall level is similar between the two locations (700 and 900 mm) and makes them medium rainy places. Rainfall therefore has little influence on the composition of the EOs. The same goes for solar exposure since in the months of collection in both locations the presence of sunlight is very significant (about 14 h of light on average) and also influences the composition of the EOs to a lesser extent.

The PCA analysis of the variability in the chemical composition of the four EOs confirmed data from literature on the combined effects of geographical factors and development stages on the phytochemical profile of *A. absinthium* EO. Furthermore, in the case of EOs obtained from plants growing in Ogliastro Cilento (Southern Italy), their chemical composition appeared to be more influenced by the phenological phase compared to plants growing in Cogne (Northwest Italy). Finally, as regard to the EOs from Cogne, our results agree with a previous work [52], which reported that in the western Alpine arc (Northern Italy) the most important chemotype, above 1000 m, was the myroxide type. Despite the relatively small sample size and limited geographical range, the encouraging results obtained serve as a basis for future studies that may be aimed at expanding the sample and including different territorial contexts to enrich the interesting disagreement of morphological and chemical variability.

The results on the chemical variability found among the EOs may suggest possible applications in the pharmaceutical and aromatherapy sectors [2]. A hypothetical selection of plant material intended for one specific sector should take into account the geographical origin and the phenological stage at the time of collection, with a view to rational and functional valorisations. The samples collected in Cogne are characterized by large quantities of myroxide and germacrene D, sesquiterpenes known for their anti‐inflammatory and antimicrobial activities [56, 57, 58], β‐phellandrene and β‐myrcene, known for their anti‐inflammatory, antimicrobial and analgesic properties [59, 60, 61] are instead the main compounds of the EO Ogliastro‐F. Finally, davanone, with antioxidant and cytotoxic properties [62, 63] is contained in high quantities in the EO Ogliastro‐V. The phytochemical diversity found suggests a possible use of the EOs for specific problems (e.g., respiratory or skin diseases, inflammatory conditions or as dermocosmetics) where it is important that the choice of the best EO is based on the evaluation of its chemical composition.

## 3 Conclusions

The available scientific literature shows how environmental conditions have a strong influence on the secondary metabolism of plants, particularly on aromatic ones. The leaf dimension and composition of the EO of A*. absinthium* growing in Northern and Southern Italy were significantly affected by both environmental factors and phenological stages. In particular, plants growing in Cogne (Northern Italy), where there is an Alpine climate, showed larger, more developed and deeply incised leaves and EOs belonging to the myroxide chemotype. On the contrary, plants growing in Ogliastro Cilento (Southern Italy), where there is a Mediterranean climate, had smaller leaves and EOs of a mixed chemotype. Finally, EOs from Cogne‐V and Cogne‐F showed only qualitative variations, while the EOs Ogliastro‐V and Ogliastro‐F differed both qualitatively and quantitatively. The results of this study point out that optimal harvesting conditions, considering both geographical origin and harvest time, are very useful if one wants to obtain the maximum concentration of desired compounds responsible for certain properties. Practically, these data could be used for a more effective management of the available natural resources and to promote a more rational harvesting. Subsequent studies plan to expand the sample size and include different territorial contexts, to validate and generalize the results obtained in the present work.

## 4 Experimental Section

### 4.1 Plant Material

In both locations (Cogne and Ogliastro Cilento), samples were taken from five plants for each population, at different stages (vegetative and flowering), to obtain the starting plant material.

Samples of *A. absinthium* were harvested in June 2023, during the flowering stage, and at the end of May 2024, when the plant had not yet bloomed (at vegetative stage) (Figure 1A), in the hamlet of Lillaz (Cogne, Aosta Valley, Italy), on the Northwestern Alps, at 1560 m a.s.l. The species was identified by Dr. Andrea Mainetti and a voucher specimen, labelled CS189005, was deposited at the herbarium of the Alpine Botanical Garden ‘Paradisia’, managed by the Gran Paradiso National Park, which is located in Valnontey valley in the municipality of Cogne (Aosta, Italy). The sampling site is located near the locality of Ronc, just before the hamlet of Lillaz, on a dry slope at 1600 m a.s.l., facing southwest. It is characterized by a xerothermic grassland dominated by *Festuca valesiaca*, interspersed with patches of *Juniperus sabina*, a characteristic vegetation community of the Cogne Valley. The valley has an endalpic climate with mean annual precipitation of about 700 mm and mean annual temperature of +4.1°C (mean values of Lillaz weather stations, at 1700 m a.s.l.). The endalpic alpine continental climate is characterized by cold winters and mild summers [64]. The valley's climatic conditions vary with altitude, influencing vegetation distribution. Xerothermic conditions can be found on south‐facing slopes, favouring steppe‐like vegetation, while humid and cooler microclimates persist in shaded areas and along watercourses. This climatic heterogeneity contributes to the region's rich biodiversity, supporting a mosaic of plant communities adapted to different environmental conditions.

Samples of *A. absinthium* were collected in May 2024, during the vegetative stage, and in July 2024, during blooming, in a private field near the municipality of Ogliastro Cilento (Salerno, Campania Region, Italy), at about 360 m a.s.l. The species was identified by Prof. Vincenzo De Feo and a voucher specimen, labelled DF245/2024, was deposited at the herbarium of the University of Salerno. The collection site is a hilly area 8 km from the sea that has environmental characteristics typical of the thermo‐Mediterranean zone. The climate is typically Mediterranean, with short, hot, muggy, dry and mostly clear summers; long, cold, wet and partially cloudy winters. Temperatures generally vary throughout the year between 7°C and 31°C, rarely falling below 2°C or exceeding 34°C. The average annual temperature is around +15°C. The climate is sub‐humid, with average annual precipitation of around 900 mm (average data from the meteorological station of Capaccio, at 450 m a.s.l.). Sunlight is at its strongest between April and September, with an average of 12–15 h of sunshine per day, while between October and March it drops to 9–11 h [65].

All plant samples were cleaned of soil residues and air‐dried for 7 days. Then, they were stored in containers away from light and humidity until distillation.

### 4.2 Macro‐Micromorphological Analyses

The macromorphological characteristics of fresh leaves of both samples were first observed with a LEICA M205 C stereomicroscope (Leica Microsystems, Wetzlar, Germany). Thereafter, sections from leaves, petiole, stem, and inflorescence were analysed by light microscopy to define their micro‐morphological features. Vegetative organs were handmade cross‐sectioned using a double‐edged razor blade, while the flowers were cut lengthwise with fine tweezers and gently placed on a slide. Observations were carried out on fresh sections or after fixation of the samples in a 70% ethanol–FineFix solution (Milestone SRL, Sorisole, Bergamo, Italy) for 24 h at 4°C. Some sections were cleared with an aqueous solution of chloral hydrate and treated with a chloral hydrate–glycerol solution to prevent crystallization of the reagent during the observation of the slides [66]. The following histochemical stains were tested: phloroglucinol‐HCl (Merck, Darmstadt, Germany) for lignin, and Sudan III, Sudan Black and Fluorol Yellow 088 (Merck, Darmstadt, Germany) to detect the presence of lipophilic substances [67]. Observations were made with a Leica DM 2000 fluorescence microscope equipped with an H3 filter (excitation filter BP 420–490 nm) (Leica Microsystems, Wetzlar, Germany) and a ToupCam Digital Camera, CMOS Sensor 3.1 MP resolution (ToupTek Photonics, Hangzhou, China).

To achieve an in‐depth characterization of the micro‐morphological and anatomical features, aerial portions were also analysed by SEM. After fixation, samples were dehydrated in solutions with increasing ethanol content (70%, 80%, 90%, and 100%) for 1 h each and then critical point‐dried using liquid carbon dioxide (CO_2_) (K850CPD 2 M, Strumenti S.r.l., Roma, Italy). Finally, dried samples were mounted on aluminium stubs using two‐sided adhesive carbon tape and covered with a 10 nm layer of gold particles. The examination was performed under a VEGA3‐Tescan‐type LMU microscope (Tescan USA Inc., Cranberry Twp, PA, USA), operating at an accelerating voltage of 20 kV.

### 4.3 Extraction of the EOs

Aerial parts of the plant were steam‐distilled for 2 h, according to the method reported by the *European Pharmacopoeia* [68]. The EOs were solubilized in *n*‐hexane, filtered over anhydrous sodium sulphate, and stored under N_2_ at +4°C in the dark until tested and analysed.

### 4.4 GC and GC/MS Analyses

The composition of the EOs was achieved by GC and GC‐MS. GC analyses were performed using a Perkin‐Elmer Sigma 115 gas chromatograph equipped with a flame ionization detector (FID), on a non‐polar HP‐5 MS capillary column of fused silica (30 m × 0.25 mm; 0.25 µm film thickness). The operating conditions were as follows: injector and detector temperatures, 250°C and 290°C, respectively. The analysis was conducted on a scheduled basis: 5 min isothermally at 40°C; subsequently, the temperature was increased by 2°C/min until 270°C and finally it was kept in isotherm for 20 min. The analysis was also performed on an HP Innowax column (50 m × 0.20 nm; 0.25 µm film thickness). In both cases, He was used as a carrier gas (1.0 mL/min). GC‐MS analysis was performed using an Agilent 6850 Ser. II Apparatus connected to an Agilent Mass Selective Detector (MSD 5973); ionization voltage 70 V; ion multiplier energy 2000 V. The mass spectra were scanned in the range of 40–500 amu, with five scans per second. The chromatographic conditions were as above reported, and the transfer line temperature was 295°C. Most of the components were identified by comparing their Kovats indices (Ki) with those of the literature [69, 70, 71, 72] and by a careful analysis of the mass spectra compared to those of pure compounds available in our laboratory or to those present in the NIST 02 and Wiley 257 mass libraries [73]. The Kovats indices were determined in relation to a homologous series of *n*‐alkanes (C10‐C35), under the same operating conditions. For some compounds, the identification was confirmed by co‐injection with standard samples. Components' relative concentrations were calculated by peak area normalization. Response factors were not considered.

### 4.5 PCA

PCA and HCA were conducted using XLSTAT software (Addinsoft, Paris, France). PCA was employed to investigate the variance and underlying patterns within the dataset. Before analysis, the data were standardized to ensure that each variable contributed equally to the model. The analysis was based on the correlation matrix, and principal components were extracted following Kaiser's criterion, with eigenvalues greater than 1. The percentage of variance explained by each component was assessed, and the corresponding loadings were examined to identify the variables most significantly contributing to the observed patterns. HCA was applied to classify the observations based on their similarities. Clustering was carried out using Ward's method. A dendrogram was generated to represent the cluster hierarchy, and the optimal number of clusters was determined by cut‐off level.

### 4.6 SIMPER Analysis

The identification of the compounds responsible for the differentiation between the groups was obtained through a SIMPER analysis using the PAST 5.2.2 software. The analysis was performed on the matrix of the relative percentage content of the compounds in the EOs, using the Bray–Curtis distance as a measure of dissimilarity.

## Author Contributions

**Paola Malaspina**: investigation, validation, writing – original draft. **Flavio Polito**: formal analysis, investigation, validation, writing – original draft. **Andrea Mainetti**: investigation, writing – original draft. **Sana Khedhri**: formal analysis, validation, writing – original draft. **Vincenzo De Feo**: conceptualization, resources, supervision, writing – revise and editing, investigation. **Laura Cornara**: conceptualization, investigation, resources, supervision, validation, writing – revise and editing.

## Conflicts of Interest

The authors declare no conflicts of interest.

## Data Availability

The data that support the findings of this study are available from the corresponding author upon reasonable request.
